# Association of Physical Activity, Muscle Mass, Strength, and Function to Quality of Life in Older Adults

**DOI:** 10.1093/geroni/igaa057.601

**Published:** 2020-12-16

**Authors:** Murad Taani, Chi Cho, Julie Ellis

**Affiliations:** 1 University of Wisconsin-Milwaukee, Milwaukee, Wisconsin, United States; 2 University of Wisconsin Milwaukee, Milwaukee, Wisconsin, United States

## Abstract

Physical inactivity and loss of muscle mass, strength, and function are associated with negative outcomes including disability and a decline in health-related quality of life (HRQoL) among older adults. Older adults living in continuing care retirement communities (CCRCs) are at greater risk for declining physical activity and muscle outcomes compared to community-dwelling older adults. Few researchers studying the association of muscle and physical activity have examined the distinction between physical and mental HRQoL. Understanding the differential association of physical and mental HRQoL to physical activity and muscle outcomes can inform the development of useful interventions. The aim of this study was to examine the relationships between physical activity, muscle mass, strength, function and physical and mental HRQoL. Using a descriptive, correlational design, 105 older adults living in CCRCs were recruited. Light physical activity (LPA), moderate physical activity (MPA), sedentary behavior, and steps per day were assessed using ActiGraph GT3X. Appendicular skeletal muscle mass (ASMM) was assessed with bioelectrical impedance spectroscopy, handgrip strength with JAMAR Smart Hand Dynamometer, muscle function with the Short Physical Performance Battery (SPPB) test, and physical and mental HRQoL with the SF-36 questionnaire. The mean age of participants was 83 (SD=7.4). Using multiple regression models adjusted for sex and age, steps per day and SPPB score explained 38.4 % of the variance in physical HRQoL. Handgrip strength explained 8 % of the variance in mental HRQoL. These findings suggest that QoL improvement programs should include components to improve physical activity, muscle strength and function.

